# Positive-Unlabeled Learning in Implicit Feedback from Data Missing-Not-At-Random Perspective

**DOI:** 10.3390/e28010041

**Published:** 2025-12-29

**Authors:** Sichao Wang, Tianyu Xia, Lingxiao Yang

**Affiliations:** 1 KUKA Robotics China Co., Ltd., Shanghai 201702, China; wangsichao13@163.com; 2 Department of Biostatistics, School of Public Health, Peking University, Beijing 100871, China; 3 School of Artificial Intelligence, Anhui University, Hefei 230601, China; yanglingxiao@ahu.edu.cn

**Keywords:** recommender systems, implicit feedback, missing-not-at-random, positive-unlabeled learning

## Abstract

The lack of explicit negative labels issue is a prevalent challenge in numerous domains, including CV, NLP, and Recommender Systems (RSs). To address this challenge, many negative sample completion methods are proposed, such as optimizing sample distribution through pseudo-negative sampling and confidence screening in CV, constructing reliable negative examples by leveraging textual semantics in NLP, and supplementing negative samples via sparsity analysis of user interaction behaviors and preference inference in RS for handling implicit feedback. However, most existing methods fail to adequately address the Missing-Not-At-Random (MNAR) nature of the data and the potential presence of unmeasured confounders, which compromise model robustness in practice. In this paper, we first formulate the prediction task in RS with implicit feedback as a positive-unlabeled (PU) learning problem. We then propose a two-phase debiasing framework consisting of exposure status imputation, followed by debiasing through the proposed doubly robust estimator. Moreover, our theoretical analysis shows that existing propensity-based approaches are biased in the presence of unmeasured confounders. To overcome this, we incorporate a robust deconfounding method in the debiasing phase to effectively mitigate the impact of unmeasured confounders. We conduct extensive experiments on three widely used real-world datasets to demonstrate the effectiveness and potential of the proposed methods.

## 1. Introduction

Positive-unlabeled (PU) learning addresses scenarios where training data comprises only labeled positive entries and unlabeled entries, the latter of which may be a mixture of unobserved true negatives or unrecognized positives [1,2]. Such challenges are commonly seen in various real-world machine learning tasks [3,4,5,6], like in computer vision [7], natural language processing [8], and healthcare analytics [9]. Ignoring such mixture and simply treating all unlabeled ones as negative entries would lead to biased estimation and hurt the performance [10].

Among these domains, a recommender system (RS), which plays a key role in many modern websites and mobile applications by filtering out information that may be of interest to users, is another representative scenario [11,12,13]. In general, there are two types of user feedback in RS for training the prediction model, explicit feedback and implicit feedback. The explicit feedback (e.g., ratings) directly reflects users’ preferences but requires additional effort and cost to collect, making it often inaccessible in practice [14]. In contrast, the implicit feedback (e.g., click or purchase) only partially reveals user preferences but can be easily obtained by just recording user behavior logs [11,15,16,17], but users usually interact with what interest them only.

Studying a recommendation model with implicit feedback is challenging, primarily due to two core issues: *missing-negative-feedbak* (MNF) and the problem of *missing-not-at-random* (MNAR) [18,19]. For example, in the field of information retrieval, assume a click denotes positive feedback, then unlabeled ones cannot know whether unclicked events are negative feedback (not relevant) or unexposed feedback, i.e., the true negative and potentially positive feedback are entangled together in the unclicked events [20]. In addition, since the users tend to click the more preferred items (selection bias) and the system prefers to recommend popular items (exposure bias), the user’s feedback is not missing completely at random. This MNAR problem is further intensified by unmeasured confounders, hidden factors that affect both exposure and feedback [21]. For instance, a user’s latent financial status may sway preference towards affordable items and simultaneously affect their exposure via platform ranking policies. Similarly, an item’s inherent but unmeasured popularity can create a feedback loop where it is both preferred more and shown more frequently. Such confounders create spurious correlations, leading to over-recommending popular or low-price items. Ignoring this problem would incur bias and lead to sub-optimal performance [22,23,24]. Our work addresses those biases while accounting for these hidden factors under the MNAR setting.

Plenty of literature focus on addressing such MNF issues in RS. For instance, some works treat unclicked events as negatives and assign them lower weights [20,25,26]; others sample a few representative items from unclicked events to approximate true negatives [27,28,29]. Until [30], the study of RS implicit feedback was formally formulated as a PU learning problem. In recent years, there has been increasing interest in tackling MNF together with MNAR problems. Based on causal inference, refs. [14,31] propose a new ideal loss and an inverse propensity score (IPS) method for unbiased learning. Refs. [32,33] extend IPS by combining a joint-learning method and handling the bias in missing feedback, respectively. Other than selection bias in implicit feedback, refs. [18,34] further point out others, like exposure bias and bias due to sensitive privacy attributes, together with their corresponding debiasing methods.

Despite these advances of existing debiasing methods for the PU/MNF problem, we identify two critical limitations, not just for implicit feedback in RS: (1) Previous studies have not fully explored the MNAR problem. Works within the PU framework, like [35,36], were limited to addressing the selected-at-random (SAR, or equivalently, missing-at-random, MAR) setting. Furthermore, they solely presented IPS estimators, with no exploration of double robust (DR) methods, a classic extension of IPS. Even those causal RS works studying the MNF issue [14,32,33] addressed the MNAR problem via an IPS estimator, and they also omitted a specification of the DR method. Given that the DR method [37] and its variants [38,39,40,41] have achieved state-of-the-art performance for explicit feedback unbiased learning, developing DR-based methods for PU scenarios with MNAR is highly desirable; (2) no existing methods explicitly addressed unmeasured confounders. While methods in [4,15,30,42] tackled biased feedback with the MNF issue, they did not consider unmeasured confounders, which are unavoidable in RS due to technical difficulties or privacy restrictions [43,44,45], etc. Our theoretical analysis shows that propensity score-based methods become biased under unmeasured confounders, making it necessary to develop targeted solutions for implicit feedback in RS.

To address these research gaps, we first formulate the implicit feedback problem as a PU learning task to enhance prediction robustness [2,4,30,35].

A key insight of this formulation is that all the previous debiasing methods for explicit feedback can be extended to implicit feedback, provided that exposure status (or equivalently, treatment) is properly imputed. This insight motivates our proposed two-phase debiasing framework, which consists of a treatment imputation phase and a debiasing phase. The treatment imputation step is treated as a one-class classification problem, and we adopt a commonly used method, Support Vector Data Description (SVDD) [46]. For the debiasing phase, beyond applying IPS/DR explicit-feedback debiasing methods given MNAR, we also account for the impact of unmeasured confounders. Notably, the proposed framework provides a flexible paradigm that seamlessly bridges the debiasing methods for explicit and implicit feedback, especially enabling DR methods for implicit feedback with the PU learning problem. Our main contributions are summarized as follows:

- We propose a two-phase debiasing framework that provides a PU learning paradigm with corresponding IPS and DR estimators to predict preference using implicit feedback.
- We reveal the risk of unmeasured confounders for implicit feedback and propose a debiasing method to robustly mitigate the bias.
- We conduct extensive experiments to validate the effectiveness of the proposed framework and methods.

## 2. Related Works

Various biases affect recommender systems [47], both in explicit feedback data [48] and implicit feedback data [14], including popularity bias [49], model selection bias [50], user self-selection bias [51], position bias [52], conformity bias [53], etc. To address these, causal inference methods have been widely adopted to develop debiasing estimators. The IPS approach and its self-normalized variant (SNIPS) were pioneering methods for debiasing explicit feedback [22]. Subsequent work introduced the DR estimator [37], which provides robustness against misspecification of either the propensity or imputation model, and has smaller variance than the IPS method [16,54]. Enhanced DR methods were later proposed, such as MRDR [38], SDR [55], TDR [56], and typically, a robust deconfounding approach (RD-IPS/RD-DR) that handles unmeasured confounders through adversarial training [57]. For a review, [47] provided a thorough discussion of recent progress on debiasing tasks in RS, and [23] established a unified causal analysis framework and formal causal definitions of various biases in RS based on a violation of assumptions adopted in causal analysis.

In parallel, implicit feedback poses a unique challenge due to the absence of a true negative label and the mixture of potential positives and negatives among unlabeled entries. Early heuristic approaches included WMF [15], which down-weights unobserved samples; BPR [42], which employs pairwise ranking; and ExpoMF [58], which incorporates exposure modeling. However, these methods lack unbiasedness guarantees under PU data, and they also discuss the additional MNAR problem. Later, propensity-based methods emerged to tackle the MNF imbalanced issue and discuss unbiasedness properties: RMF [14] presented IPS loss, UBPR further handled bias in missing feedback [59], and CJMF [32] combined it with joint learning; while [18,34,60] additionally handled MNAR due to selection bias, privacy bias and bias due to privacy attributes, respectively. However, these methods were not formalized as PU learning and did not specify the corresponding DR estimators.

WeaklyRec [30], as a tipping point, formally framed implicit RS feedback as a PU learning problem, together with PURL [61], which improved upon it via class prior estimation. They both relied on weak supervision (not propensity-based methods) and did not address any additional bias or MNAR issues. In general PU learning, two paradigms dominate [30]: (1) Re-weighting for unbiased estimation of the ideal loss, including uPU [62] and its convex/non-negative variants [2,3,6], further under the impact of imbalanced data and given selection bias; (2) Pseudo-labeling, using techniques like confidence score [63], generative models [64], clustering analysis [65], and reinforcement learning [66] to identify reliable negatives/positives from unlabeled data. Although some PU methods offered debiasing estimators with an unbiased property via propensity-based methods [3,6,35,36], most only considered MAR/SAR settings, with refs. [4,63] considering additional selection bias. In summary, the critical gap persists. Our proposed framework aims to address the following limitations: debiasing methods for the PU learning problem (not just for RS implicit feedback), a lack of formalized DR loss, and insufficient discussion on the potential impact of MNAR and unmeasured confounders.

## 3. Problem Setup

Let U={u}, I={i} and D=U×I denote the sets of users, items and user-item pairs, respectively. For each user–item pair, let $x_{u,i}$ represent its feature vector and $y_{u,i}\in \left\{0,1\right\}$ denote the implicit feedback, where $y_{u,i}=1$ or 0 indicates whether user *u* clicks item *i* or not. In the setting of implicit feedback, $y_{u,i}=1$ represents a positive feedback, but we cannot determine whether $y_{u,i}=0$ represents a true negative feedback (i.e., the user dislikes the item), or a potential positive feedback (i.e., the item is relevant but never shown to the user). To resolve this ambiguity, we introduce two *unobserved* variables, exposure status $o_{u,i}$ and relevance $r_{u,i}$. $o_{u,i}=1$ or 0 represents whether item *i* is exposed to user *u* or not; $r_{u,i}=1$ or 0 represents whether user *u* and item *i* are relevant or not. With these notations, the existing works [4,14,30,35] assume a user clicks an item *only if* the item is both exposed and relevant, leading to the below assumption:

**Assumption** **1.** *$y_{u,i}=o_{u,i}\cdot r_{u,i}$*, *for all (u,i)∈D.*

Under Assumption 1, if we intervene to set $o_{u,i}=1$, then $r_{u,i}=y_{u,i}$, i.e., the observed feedback directly reveals true relevance. Unobserved entries could be missing data with either positive labels (i.e., user and item are truly relevant) or negative labels (i.e., user and item are non-relevant). In real-world applications, each user can only interact with (e.g., clicks, views) a very limited number of items, making such missing data ubiquitous.

### PU Learning for Recommendation System

Unlike most prior literature, we formulate the implicit feedback problem using the positive-unlabeled framework following the work of [30], which is also a widely adopted framework in weak- or semi-supervised learning [4,6,35,36]. In a PU learning problem, a full dataset D consists of entries arising from two marginal distributions: $p_{P}\left(x\right)=p\left(x|r=1\right)$, the distribution of features for truly relevant (positive) user–item pairs, and $p_{N}\left(x\right)=p\left(x|r=0\right)$, the distribution of features for truly irrelevant (negative) user–item pairs. The overall data distribution of the full dataset D can be expressed as a mixture of these two distributions:

(1) $$p_{D}\left(x\right)=\pi \cdot p_{P}\left(x\right)+\left(1-\pi \right)\cdot p_{N}\left(x\right),$$

where π=P(r=1|x) denotes the class prior of positive label.

Notably, the true relevance *r* is hidden, and only the partially labeled *y* is observed, and the implicit feedback dataset follows the core PU property, i.e., all observed labels are positive feedback and correspond to true positives, but the unlabeled ones are mixed with true negatives and unexposed positives. Therefore, the goal of PU learning in this paper is to train a binary classification model f(x) that predicts the true relevance $r_{u,i}$. Let ${\hat{r}}_{u,i}$ be the prediction of $r_{u,i}$.

Owing to this reformulation of the data distribution, we can define the ideal loss for implicit feedback, which matches the ideal loss used in explicit feedback studies if we could observe all pairs of users’ preferences [2]. This ideal loss decomposes as

(2) $$E\left[L_{ideal}\right]=E_{p_{D}}\left[\delta_{r}\left(\hat{r}\right)\right]=\pi E_{p_{P}}\left[\delta_{1}\left(\hat{r}\right)\right]+\left(1-\pi \right)E_{p_{N}}\left[\delta_{0}\left(\hat{r}\right)\right]$$

where $\delta_{r}\left(\hat{r}\right)=l\left(r,\hat{r}\right)$, l(·) is a pre-specified loss, e.g., the cross-entropy loss $l\left(r_{u,i},{\hat{r}}_{u,i}\right)=r_{u,i}\log{\hat{r}}_{u,i}+\left(1-r_{u,i}\right)\log\left(1-{\hat{r}}_{u,i}\right)$. For brevity, we simplify the user-item level loss to $\delta_{u,i,r_{u,i}}$. Empirically, we aim to train a model that minimizes the risk

(3) $$L_{ideal}=\frac{1}{\left|D\right|}\sum_{(u,i)\in D}r_{u,i}\delta_{u,i,1}+\left(1-r_{u,i}\right)\delta_{u,i,0}.$$

This ideal loss aligns with those defined in explicit feedback literature if all $r_{u,i}$ were observable. In practice, however, $L_{ideal}$ is infeasible, because we can not observe all. A common workaround is to construct an unbiased estimator of $L_{ideal}$ unbiasedly, like IPS [22], but this is challenging, since classical debiasing methods for explicit feedback require true negative samples, which are unavailable in implicit feedback.

Conversely, if $o_{u,i}$ can be properly inferred, then the implicit feedback data on the exposed events $\left\{\left(u,i\right)\in D:o_{u,i}=1\right\}$ would become explicit feedback data by Assumption 1, and thus the debiasing approaches for explicit feedback can apply to implicit feedback. This insight inspires us to decompose the whole task into two phases: (1) impute the unobserved exposure status $o_{u,i}$; (2) perform debiased learning using the imputed exposure information. We detail this framework in the next section.

## 4. Proposed Method

In this section, we propose a two-phase debiasing framework for implicit feedback that provides a flexible paradigm seamlessly linking the debiasing approaches for explicit feedback and those for implicit feedback.

### 4.1. PU Learning for Implicit Feedback

For data with a PU structure, the unlabeled data distribution is a mixture of positive and negative data distribution:

(4) $$p_{U}=\pi p_{P}\left(x\right)+\left(1-\pi \right)p_{N}\left(x\right).$$

In practice, however, we only observe the label *y* rather than the true relevance *r*. Therefore, we alternatively calculate $\delta_{y}\left(\hat{r}\right)=l\left(y,\hat{r}\right)$, simplifying the notation of user–item level loss to $\delta_{u,i,y_{u,i}}$. Based on this formulation, the mixed negative predicted errors can be expressed in terms of the unlabeled and positive entries:

(5) $$\left(1-\pi \right)E_{p_{N}}\left[\delta_{0}\left(\hat{r}\right)\right]=E_{p_{U}}\left[\delta_{0}\left(\hat{r}\right)\right]-\pi E_{p_{P}}\left[\delta_{0}\left(\hat{r}\right)\right].$$

We subsequently derive a surrogate of the ideal risk expressed in terms of PU implicit feedback,

(6) $$E_{p_{D}}\left[L_{PU}\right]=\pi \left(E_{p_{P}}\left[\delta_{1}\left(\hat{y}\right)\right]-E_{p_{P}}\left[\delta_{0}\left(\hat{r}\right)\right]\right)+E_{p_{U}}\left[\delta_{0}\left(\hat{r}\right)\right].$$

### 4.2. Two-Phase Debiasing Framework

**Figure 1:**
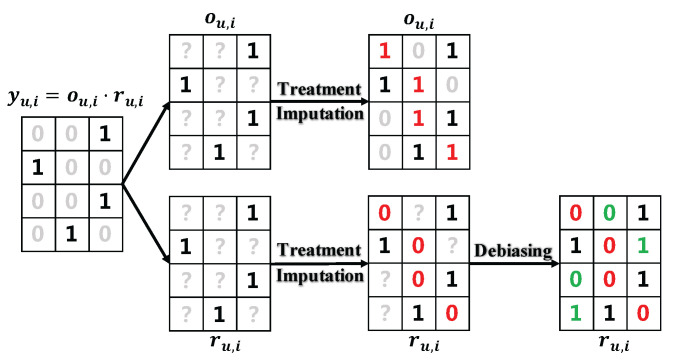
Two-phase debiasing framework for implicit feedback. **1**: Observed positive label, **0**: Unlabeled entry; **?**: Entry with unknown status for explicit feedback; **1**/**0**: Imputed label after treatment imputation; **1**/**0**: Predicted label after debiasing.

The proposed two-phase debiasing framework consists of two stages: the treatment imputation phase and the debiasing phase. Figure 1 presents the structure of the proposed framework.

In the first treatment imputation phase, unlike previous studies [25,27] where negative sampling methods of $y_{u,i}$ were used, we aim to do *positive* sampling of $o_{u,i}$. Then for the exposed events $\left\{\left(u,i\right)\in D:o_{u,i}=1\right\}$, we know the exact value of $r_{u,i}=y_{u,i}$ according to Assumption 1. This leads to the data structure similar to explicit feedback, as shown in the middle of Figure 1. The advantages of this phase is three-fold: (1) it provides a flexible paradigm that can link the debiasing methods in explicit feedback and those in implicit feedback, whereas existing methods like [14,18,32] did not have DR losses due to the lack of treatment. As a result, they need to handle the missingness of $r_{u,i}$ and $o_{u,i}$ simultaneously; (2) it provides a chance for obtaining more accurate estimates of propensities; (3) it gives a more accurate imputation of $r_{u,i}$ through the fact that $y_{u,i}=o_{u,i}r_{u,i}$.

After imputing the treatment, although we can know the explicit values of $r_{u,i}$ for exposed events, the values of $r_{u,i}$ for unexposed events are still missing. Ignoring this missing problem will incur bias and lead to sub-optimal performance. Thus, it is necessary to consider the debiasing phase. Due to the treatment imputation phase, all previous debiasing methods for explicit feedback, such as DR-JL [37] and MRDR [38], can be applied in parallel to implicit feedback. More importantly, we also consider the existence of unmeasured confounders, which invalidate most existing debiasing methods and have not been studied in the literature of implicit feedback.

Another potential strength is accounting for unmeasured confounders in the debiasing phase. In the treatment imputation phase, it is inevitable to introduce bias induced by inaccurate imputation, leading to a degradation of prediction performance. In such a task, we could take the bias as the influence of unmeasured confounders, which is intuitively reasonable. On the other hand, unmeasured information should not dominate the observed ones. Thus, while the imputation phase may introduce some bias, it provides good starting points for the debiasing phase.

### 4.3. Treatment Imputation Phase

The treatment imputation phase targets the problem of missing treatment. Missing treatment means missing $o_{u,i}$ and, as a result, the inability to identify the true relevance of negative samples in exposed events. As mentioned in Section 1, one of the biggest differences between explicit and implicit feedback is the mixture of truly negative and potentially positive feedback. Fortunately, suppose we have the true value of $o_{u,i}$, we can identify the true negative samples on the exposed events according to Assumption 1, and this inspires the first phase, the treatment imputation.

Since, according to Assumption 1, we have only samples with $o_{u,i}=1$ for the group of $y_{u,i}=1$ and we want to distinguish the value of $o_{u,i}$ for the observed negative samples, treatment imputation is a one-class classification (OCC) problem. A range of well-established OCC methods can be utilized to solve this problem: one-class support vector machines (OC-SVM) [67], support vector data description (SVDD) [46], density-based methods [68], principal component analysis (PCA) [69], (one-class) K-means [70], autoencoder-based reconstruction methods [71], density-based isolation forests [72], etc. These methods all aim to capture the characteristic pattern of positive samples to classify unlabeled data into the labeled class or the unlabeled class. In this work, we adopt SVDD, one of the most widely used OCC methods, to deal with this problem. The idea behind SVDD is to find an optimal hyper-sphere of radius *R* that includes as many positive samples as possible. This hyper-sphere has clear physical interpretations. Its center *a* represents the “typical feature vector” of exposed user–item pairs, such as common attributes of items that are recommended and interacted with, while the radius *R* quantifies the similarity threshold for exposure. This transparency allows tracing why an unlabeled sample is imputed as a true negative.

In our framework, we first train an SVDD classifier and then predict the exposure status for the negative samples. If a negative sample is in this hyper-sphere, then we label it with $o_{u,i}=1$, and set $o_{u,i}=0$ for those outside the hyper-sphere. Then, for the newly labeled samples with $o_{u,i}=1$, we know $r_{u,i}=0$ according to Assumption 1, and we can form a new training set using these samples as well as the observed positive samples, which completes the phase of treatment imputation. Essentially, the SVDD classifier assumes that user–item pairs with similar features should have similar exposure status, which is similar to the idea of collaborative filtering used in model recommender systems, where similar users are assumed to have similar behavior.

### 4.4. Debiasing Phase

As discussed in Section 3, debiasing approaches previously developed for explicit feedback can be applied to implicit feedback after the treatment has been imputed. To accommodate a wider range of application scenarios, we consider two cases in the debiasing phase: the absence of unmeasured confounders and the presence of unmeasured confounders.

#### 4.4.1. In the Absence of Unmeasured Confounders

When no unmeasured confounders are present, we develop our method based on the idea of propensity-based approaches for learning PU data with missing-at-random (MAR) or selected-at-random (SAR) labeling mechanisms [14]. In PU learning, we define the propensity score as the positively labeled probability conditioned on the true positives:

(7) $$p_{u,i}=P\left(y_{u,i}=1|r_{u,i}=1,x_{u,i}\right),$$

since the exposure indicator is not fully observable, but we will later show that it is equal to the normal propensity defined in the causal inference framework under certain conditions.

Recall that for explicit feedback training, the *inverse propensity score* (IPS) [22] and *doubly robust* (DR) methods [37,38,73] are two main debiasing strategies to address the MNAR problem. However, their formulation cannot be directly applied when working with implicit feedback that exhibits the PU problem, because the probability of labeling negative examples is zero [35], i.e., the exposure indicator is not fully observed [14]. Thus, we need to adjust the weighting strategy. Alternatively, the key insight is that among true positives, we expect $1/p_{u,i}$ to be labeled, while $\left(1/p_{u,i}-1\right)$ true positives remain unexposed and thus unlabeled, and true negatives are not labeled. Therefore, based on Equation (6) and Assumption 1, the IPS loss is formulated as

$$\begin{array}{cc} L_{IPS}\; & =\frac{1}{\left|D\right|}\sum_{(u,i)\in D}y_{u,i}\left[\frac{1}{{\hat{p}}_{u,i}}\delta_{u,i,1}+\left(1-\frac{1}{{\hat{p}}_{u,i}}\right)\delta_{u,i,0}\right]+\left(1-y_{u,i}\right)\delta_{u,i,0}\\ \; & =\frac{1}{\left|D\right|}\sum_{(u,i)\in D}\frac{y_{u,i}}{{\hat{p}}_{u,i}}\delta_{u,i,1}+\left(1-\frac{y_{u,i}}{{\hat{p}}_{u,i}}\right)\delta_{u,i,0}. \end{array}$$

As discussed in Section 1, some studies of PU learning have shown the corresponding IPS estimator, but have not adequately addressed it specifically for debiasing implicit feedback in recommender systems.

We define $\delta_{u,i}^{imp}=l\left(r_{u,i}^{imp},{\hat{r}}_{u,i}\right)$ as the imputed loss function, where $r_{u,i}^{imp}$ is the imputed value for $r_{u,i}$ provided by an imputation model. The corresponding loss can then be decomposed as

(8) $$E_{p_{D}}\left[L_{DR}\right]=\pi \left(E_{p_{P}}\left[\delta_{1}\left(\hat{r}\right)-\delta^{imp}\right]-E_{p_{P}}\left[\delta_{0}\left(\hat{r}\right)-\delta^{imp}\right]\right)+E_{p_{U}}\left[\delta_{1}\left(\hat{r}\right)-\delta^{imp}\right]+E_{p_{D}}\left[\delta^{imp}\right].$$

by Equations (1) and (4). Note that the imputation loss $\delta_{u,i}^{imp}=l\left(r_{u,i}^{imp},\hat{r}\right)$ should not be canceled out here, and we mark it as $\delta_{u,i,{\hat{r}}_{u,i}}^{imp}$ to avoid confusion.

Unlike IPS, the DR loss has not been discussed in the setting of PU learning before, and we propose to construct it as follows

$$\begin{array}{cc} L_{DR} & =\frac{1}{\left|D\right|}\sum_{(u,i)\in D}y_{u,i}\left[\frac{1}{{\hat{p}}_{u,i}}\left(\delta_{u,i,1}-\delta_{u,i,1}^{imp}\right)+\left(1-\frac{1}{{\hat{p}}_{u,i}}\right)\left(\delta_{u,i,0}-\delta_{u,i}^{imp}\right)\right]\\ \; & \;+\left(1-y_{u,i}\right)\left(\delta_{u,i,0}-\delta_{u,i,0}^{imp}\right)+\delta_{u,i}^{imp}\\ \; & =\frac{1}{\left|D\right|}\sum_{(u,i)\in D}\left[\frac{y_{u,i}}{{\hat{p}}_{u,i}}\left(\delta_{u,i,1}-\delta_{u,i,1}^{imp}\right)+\left(1-\frac{y_{u,i}}{{\hat{p}}_{u,i}}\right)\cdot \left(\delta_{u,i,0}-\delta_{u,i,0}^{imp}\right)+\delta_{u,i}^{imp}\right]. \end{array}$$

Note that the imputation loss $\delta_{u,i}^{imp}$ should not be canceled out here, since it corresponds implicitly to a label $y_{u,i}$, which cannot co-occur for one piece of user–item observed data. It is well known that the unbiasedness of both IPS and DR relies on the assumption of no unmeasured confounders, i.e.,

**Assumption** **2.** *$o_{u,i}⊥\;\;\;⊥r_{u,i}|x_{u,i}$.*

Under Assumption 2, from the perspective of causal inference for explicit feedback, $L_{IPS}$ is unbiased with respect to $L_{ideal}$ if ${\hat{p}}_{u,i}=p_{u,i}$, and $L_{DR}$ is unbiased if either ${\hat{p}}_{u,i}=p_{u,i}$ or $\delta_{u,i}^{imp}=\delta_{u,i}$ [23,57]. In addition, we note that the existing propensity score-based debiasing methods [14,18] for *implicit feedback* also rely on Assumptions 1 and 2. This is because they invoke the following assumption

(9) $$P\left(y_{u,i}=1|x_{u,i}\right)=P\left(r_{u,i}=1|x_{u,i}\right)\cdot P\left(o_{u,i}=1|x_{u,i}\right);$$

otherwise, $P(o_{u,i}r_{u,i}=1|x_{u,i})$ is not equal to $P\left(r_{u,i}=1|x_{u,i}\right)\cdot P\left(o_{u,i}=1|x_{u,i}\right)$ in general. In addition, according to Assumptions 1 and 2, we also have

(10) $$p_{u,i}=P\left(y_{u,i}=1|r_{u,i}=1,x_{u,i}\right)=P\left(o_{u,i}=1|r_{u,i}=1,x_{u,i}\right)=P\left(o_{u,i}=1|x_{u,i}\right),$$

which matches the propensity definition in the causal recommendation framework. Then

(11) $$P\left(y_{u,i}=1|x_{u,i}\right)=\pi_{u,i}\cdot p_{u,i}.$$

**Proposition** **1.** *In the absence of unmeasured confounders, under Assumptions 1 and 2, the IPS loss of implicit feedback is unbiased if ${\hat{p}}_{u,i}$ accurately estimates $p_{u,i}$.*

**Proof.**  Denote $\pi_{u,i}=P\left(r_{u,i}=1|x_{u,i}\right)$. Under Assumptions 1 and 2, we have Equation (11) and E[Y=1|x]=π·p, therefore

$$\begin{array}{cc} E_{o}\left[L_{IPS}\right]) & =\frac{1}{n}\sum_{i=1}^{n}\pi_{u,i}p_{u,i}\left(\frac{1}{{\hat{p}}_{u,i}}\delta_{u,i,1}+\left(1-\frac{1}{{\hat{p}}_{u,i}}\right)\delta_{u,i,0}\right)+\left(1-\pi_{u,i}p_{u,i}\right)\delta_{0}\left({\hat{y}}_{u,i}\right)\\ & =\frac{1}{n}\sum_{i=1}^{n}\pi_{u,i}\frac{p_{u,i}}{{\hat{p}}_{u,i}}\delta_{1}\left({\hat{y}}_{u,i}\right)+\left(1-\pi_{u,i}\frac{p_{u,i}}{{\hat{p}}_{u,i}}\right)\delta_{u,i,0} \end{array}$$

which equals the expectation of ideal loss (Equation (2)) when $\hat{p}=p$. □

**Proposition** **2.** *In the absence of unmeasured confounders, under Assumptions 1 and 2, the DR loss of implicit feedback is unbiased if either $\delta_{u,i}^{imp}$ accurately estimates $\delta_{u,i}$ or $\hat{p}\left(x_{u,i}\right)$ accurately estimates $p_{u,i}$.*

**Proof.**  Similarly, if there is no unmeasured confounding and the relationship between explicit and implicit feedback is $y_{u,i}=o_{u,i}\cdot r_{u,i}$,

$$\begin{array}{cc} E_{o}\left[L_{DR}\right]) & =E_{o}[\frac{1}{\left|D\right|}\sum_{(u,i)\in D}\pi_{u,i}\frac{p_{u,i}}{{\hat{p}}_{u,i}}\left(\delta_{u,i,1}-\delta_{u,i,1}^{imp}\right)+\left(1-\pi_{u,i}\frac{p_{u,i}}{{\hat{p}}_{u,i}}\right)\cdot \left(\delta_{u,i,0}-\delta_{u,i,0}^{imp}\right)\\ & \;+\pi_{u,i}\delta_{u,i,1}^{imp}+\left(1-\pi_{u,i}\right)\delta_{u,i,0}^{imp}] \end{array}$$

where the expectation of $\delta_{u,i}^{imp}$ also implicitly corresponds to a relevance $r_{u,i}$, and its expectation is similar to that of the ideal loss. The bias of $L_{DR}$ equals to

$$\begin{array}{cc} \mathrm{Bias}(L_{DR}) & =E_{o}\left[L_{DR}\right]-E_{o}\left[L_{ideal}\right]\\ & =\frac{1}{n}\sum_{i=1}^{n}\pi_{u,i}\left(\frac{p_{u,i}}{{\hat{p}}_{u,i}}-1\right)\left(\delta_{u,i,1}-\delta_{u,i,1}^{imp}\right)+\pi_{u,i}\left(1-\frac{p_{u,i}}{{\hat{p}}_{u,i}}\right)\left(\delta_{u,i,0}-\delta_{u,i,0}^{imp}\right). \end{array}$$

Therefore, the bias reduces to zero when $\hat{p}=p$ or $\delta^{imp}=\delta$, showing the double robustness property. □

Moreover, a variance analysis for the IPS estimator was not conducted in a previous study on positive-unlabeled learning [35,74], let alone the DR estimator. Thus, we provide the variance of the IPS and DR estimators in the following proposition.

**Proposition** **3.** *In the absence of any unmeasured confounder, under Assumptions 1 and 2, the variance of the IPS loss of implicit feedback is*

(12) $$V\left[L_{IPS}\right]=\frac{1}{\left|D\right|}\sum_{(u,i)\in D}\frac{\pi_{u,i}p_{u,i}\left(1-\pi_{u,i}p_{u,i}\right)}{{\hat{p}}_{u,i}^{2}}{\left(\delta_{u,i,1}-\delta_{u,i,0}\right)}^{2}.$$

**Proof.**  First, we define $Z_{u,i}=y_{u,i}\left[\frac{1}{{\hat{p}}_{u,i}}\delta_{u,i,1}+\left(1-\frac{1}{{\hat{p}}_{u,i}}\right)\delta_{u,i,0}\right]+\left(1-y_{u,i}\right)\delta_{u,i,0}$. Subsequently, $V\left[Z_{u,i}\right]$ can be written as

$$V\left(X_{u,i}\right)=\underset{\left(a\right)}{\underset{︸}{E\left[{\left(Z_{u,i}\right)}^{2}\right]}}-\underset{\left(b\right)}{\underset{︸}{{\left(E\left[Z_{u,i}\right]\right)}^{2}}}$$

For simplicity, we drop the subscript (u,i) in the following proof. Under Assumption 1 and 2, we have y∼Ber(πp). Therefore,

$$\begin{array}{cc} Z^{2} & =\frac{y^{2}}{{\hat{p}}^{2}}\delta_{1}^{2}+2\frac{y}{\hat{p}}\left(1-\frac{y}{\hat{p}}\right)\delta_{1}\delta_{0}+{\left(1-2\frac{y}{\hat{p}}\right)}^{2}\delta_{0}^{2}\\ \; & =\frac{y}{{\hat{p}}^{2}}\delta_{1}^{2}+2\left(\frac{y}{\hat{p}}-\frac{y}{{\hat{p}}^{2}}\right)\delta_{1}\delta_{0}+\left(1-2\frac{y}{\hat{p}}+\frac{y}{{\hat{p}}^{2}}\right)\delta_{0}^{2} \end{array}$$

where $o^{2}=o$. Leading to

$$\left(b\right)=\delta_{0}^{2}+2\frac{\pi p}{\hat{p}}\left(\delta_{1}\delta_{0}-\delta_{0}^{2}\right)+\frac{\pi p}{{\hat{p}}^{2}}\left(\delta_{1}^{2}+\delta_{0}^{2}-2\delta_{1}\delta_{0}\right).$$

Next, (a) is calculated as

$$\left(a\right)={\left(\frac{\pi p}{\hat{p}}\delta_{1}+\left(1-\frac{\pi p}{\hat{p}}\right)\delta_{0}\right)}^{2}={\left(\frac{\pi p}{\hat{p}}\right)}^{2}\delta_{1}^{2}+{\left(1-\frac{\pi p}{\hat{p}}\right)}^{2}\delta_{0}^{2}+2\frac{\pi p}{\hat{p}}\left(1-\frac{\pi p}{\hat{p}}\right)\delta_{1}\delta_{0}.$$

Therefore,

$$V\left(Z_{u,i}\right)=\left(a\right)-\left(b\right)=\frac{\pi_{u,i}p_{u,i}\left(1-\pi_{u,i}p_{u,i}\right)}{{\hat{p}}_{u,i}^{2}}{\left(\delta_{u,i,1}-\delta_{u,i,0}\right)}^{2}.$$

Since $\left\{Z_{u,i}\right\}$ is a linear combination of *o* and each $Z_{u,i}$ is an independent random variable, we have $V\left[L_{IPS}\right]=\frac{1}{\left|D\right|}\sum_{(u,i)\in D}V\left[Z_{u,i}\right]$. □

Similarly, we show the variance of the DR estimator in the following proposition.

**Proposition** **4.** *In the absence of any unmeasured confounder, under Assumptions 1 and 2, the variance of the DR loss of implicit feedback is*

(13) $$V\left[L_{DR}\right]=\frac{1}{\left|D\right|}\sum_{(u,i)\in D}\frac{\pi_{u,i}p_{u,i}\left(1-\pi_{u,i}p_{u,i}\right)}{{\hat{p}}_{u,i}^{2}}{\left[\left(\delta_{u,i,1}-\delta_{u,i,1}^{imp}\right)-\left(\delta_{u,i,0}-\delta_{u,i,0}^{imp}\right)\right]}^{2}.$$

**Proof.**  Similarly, we define $Z_{u,i}=\frac{y_{u,i}}{{\hat{p}}_{u,i}}\left(\delta_{u,i,1}-\delta_{u,i,1}^{imp}\right)+\left(1-\frac{y_{u,i}}{{\hat{p}}_{u,i}}\right)\cdot \left(\delta_{u,i,0}-\delta_{u,i,0}^{imp}\right)+\delta_{u,i}^{imp}$. Note that $E\left(\delta^{imp}\right)=r\delta_{1}^{imp}+\left(1-r\right)\delta_{0}^{imp}$. For simplicity, we omit the subscript (u,i) and let

$$C=\delta^{imp}+\left(\delta_{0}-\delta_{0}^{imp}\right),\;D=\frac{1}{\hat{p}}\left[\left(\delta_{1}-\delta_{1}^{imp}\right)-\left(\delta_{0}-\delta_{0}^{imp}\right)\right].$$

So Z=C+oD and $Z^{2}=C^{2}+2CoD+oD^{2}$. We further denote a term

$$C^{\prime }=\left[\pi \delta_{1}^{imp}+\left(1-r\right)\delta_{0}^{imp}\right]+\left(\delta_{0}-\delta_{0}^{imp}\right)=\delta_{0}+\pi \left(\delta_{1}^{imp}-\delta_{0}^{imp}\right),$$

Now the first and second moments are

$$\begin{array}{cc} E_{o}\left[Z\right] & =C^{\prime }+\pi pD=\delta_{0}+\pi \left(\delta_{1}^{imp}-\delta_{0}^{imp}\right)+\frac{\pi p}{\hat{p}}\left[\left(\delta_{1}-\delta_{1}^{imp}\right)-\left(\delta_{0}-\delta_{0}^{imp}\right)\right].\\ E_{o}\left[Z^{2}\right] & ={\left(C^{\prime }\right)}^{2}+2C^{\prime }\pi pD+\pi pD^{2}. \end{array}$$

Then, the variance (with respect to *o*) is

$$V_{o}\left(Z\right)=E_{o}\left[Z^{2}\right]-{\left(E_{o}\left[Z\right]\right)}^{2}=\pi p\left(1-\pi p\right)D^{2}.$$

Substituting *D* explicitly, we get

$$V_{o}\left(Z\right)=\frac{\pi p(1-\pi p)}{{\hat{p}}^{2}}{\left[\left(\delta_{1}-\delta_{1}^{imp}\right)-\left(\delta_{0}-\delta_{0}^{imp}\right)\right]}^{2}.$$

For the variance of DR we take the sum of the variances of those independent variables. □

#### 4.4.2. In the Presence of Unmeasured Confounders

**Figure 2:**
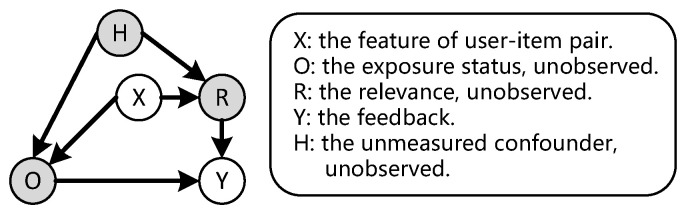
A typical causal graph for implicit feedback with unmeasured confounders *H*.

As noted by [57,75], unmeasured confounders frequently appear in real-world recommender systems. For instance, financial status may affect user preferences and feedback but remains unobservable to most recommender systems, potentially leading to over-recommendation of cheap items due to this confounding effect [57]. Figure 2 depicts this scenario for implicit feedback within the causal inference framework, where *H* denotes the unmeasured confounders affecting both *O* and *R* through the path O←H→R. In the presence of unmeasured confounders $h_{u,i}$, we specify the following Assumption 3 to substitute the previous assumption.

**Assumption** **3.** *$o_{u,i}⊥\;\;\;⊥r_{u,i}|\left(x_{u,i},h_{u,i}\right)$, $o_{u,i}⊥\;\;\;\;/\;\;\;\;⊥r_{u,i}|x_{u,i}$.*

Clearly, Assumption 3 represents a significant relaxation of Assumption 2 used in previous work [14,18]. Consequently, our framework only relies on minimal assumptions and applies to a wider range of situations. As discussed in Section 4.4.1, most existing propensity score-based methods (including IPS and DR) for both explicit feedback and implicit feedback become invalid when unmeasured confounders prevent us from disentangling the effect of $o_{u,i}$ on $y_{u,i}$. We summarize this result in Theorem 1. The proof is provided in Appendix A.

**Theorem** **1.** *In the presence of unmeasured confounders, the debiasing methods based on Equation (9) for implicit feedback are biased.*

To address the impact of unmeasured confounders, we employ the idea of adversarial training based on the robust deconfounder (RD) framework [57]. Specifically, we define the *true* propensity score in PU setting as

(14) $${\tilde{p}}_{u,i}=P\left(y_{u,i}=1|r_{u,i}=1,x_{u,i},h_{u,i}\right),$$

and refer to $p_{u,i}=P\left(y_{u,i}=1|r_{u,i}=1,x_{u,i}\right)$ the *nominal* propensity score. Considering Assumptions 1 and 3, we have

(15) $${\tilde{p}}_{u,i}=P\left(y_{u,i}=1|r_{u,i}=1,x_{u,i},h_{u,i}\right)=P\left(o_{u,i}=1|x_{u,i},h_{u,i}\right),$$

analogous to Equation (11). Furthermore, under Assumption 1, denoting $\pi_{u,i}=P\left(r_{u,i}=1|x_{u,i},h_{u,i}\right)$, and

(16) $$\begin{array}{cc} P(y_{u,i}=1|x_{u,i},h_{u,i})= & P\left(r_{u,i}=1|x_{u,i},h_{u,i}\right)\cdot P\left(o_{u,i}=1|x_{u,i},h_{u,i}\right)\\ = & {\tilde{\pi }}_{u,i}\cdot {\tilde{p}}_{u,i}. \end{array}$$

**Theorem** **2.** *In the presence of unmeasured confounders, if ${\hat{p}}_{u,i}$ accurately estimates ${\tilde{p}}_{u,i}$,*

*(a) The IPS and DR losses for explicit feedback are unbiased.*

*(b) Under Assumptions 1–3, the debiasing methods based on Equation (16) for implicit feedback are unbiased.*

Theorem 2 indicates that a true propensity score is critical to restore unbiasedness in propensity-based methods for both explicit and implicit feedback. The proof is provided in Appendix A. However, obtaining an accurate estimate of ${\tilde{p}}_{u,i}$ requires access to both measured confounders $x_{u,i}$ and unmeasured confounders $h_{u,i}$, which is generally impossible without making stringent assumptions due to the unavailability of $h_{u,i}$.

To mitigate the impact of unmeasured confounders on the estimation of the propensity score in the IPS/DR estimators, [57] employed the approach in [76] to discuss the robustness of recommendation debiasing under unmeasured confounders. Instead of completely removing the unmeasured confounding, the RD framework seeks to find the bounds of ${\tilde{p}}_{u,i}$ under a mild assumption and then applies an adversarial learning technique by varying the propensity scores with these bounds. Without loss of generality, we assume $p_{u,i}=P\left(o_{u,i}=1|x_{u,i}\right)=\sigma \left(m\left(x_{u,i}\right)\right)$ and $\tilde{P}\left(o_{u,i}=1|x_{u,i},h_{u,i}\right)=\sigma \left(\tilde{m}\left(x_{u,i},h_{u,i}\right)\right)$, where *m* and $\tilde{m}$ are two arbitrary functions, σ(x)=exp(x)/{1+exp(x)}. The following Assumption 4 establishes the relationship between the nominal propensity score $p_{u,i}$ and the true propensity score ${\tilde{p}}_{u,i}$.

**Assumption** **4.** *$|\tilde{m}\left(x_{u,i},h_{u,i}\right)-m\left(x_{u,i}\right)|\leq \log\left(\Gamma \right)$ for Γ≥1.*

The hyper-parameter Γ measures the influence of unmeasured confounding, where Γ=1 means no influence, while a larger Γ indicates the model tolerates stronger impacts from unmeasured confounders. Under Assumption 4, we can derive the bounds of ${\tilde{w}}_{u,i}=1/{\tilde{p}}_{u,i}$ given as in Lemma 1. The proof is provided in Appendix A, following the logic of [57].

**Lemma** **1.** *Under Assumption 4, we have that*

(17) $$\begin{array}{cc} a_{u,i}\leq & {\tilde{w}}_{u,i}\leq b_{u,i},\\ a_{u,i}=1+\left(1/p_{u,i}-1\right)/\Gamma & ,\;b_{u,i}=1+\left(1/p_{u,i}-1\right)\Gamma . \end{array}$$

Lemma 1 presents an uncertain set of the true propensity score. Based on Lemma 1, we define

(18) $$W=\{W\in R_{+}^{\left|D\right|}:{\hat{a}}_{u,i}\leq w_{u,i}\leq {\hat{b}}_{u,i}\},$$

where $W={\left(w_{11},\ldots ,w_{\left|U||I\right|}\right)}^{\prime }$, ${\hat{a}}_{u,i}$ and $b_{u,i}$ are estimates of $a_{u,i}$ and $b_{u,i}$. The parameter Γ provides a mechanism to quantify assumptions about unmeasured confounding roughly. By defining explicit bounds $\left[a_{u,i},b_{u,i}\right]$ on the propensity scores, the method makes its sensitivity to unmeasured confounders quantitatively traceable, allowing practitioners to assess how recommendations might vary under different confounding scenarios.

To mitigate the influence of unmeasured confounders, the loss of RD-IPS, under Assumptions 1, 3, and 4 for the PU learning framework, is modified as

(19) $$L_{RD-IPS}=\max_{W\in W}\frac{1}{\left|D\right|}\sum_{(u,i)\in D}y_{u,i}\left[w_{u,i}\delta_{u,i,1}+\left(1-w_{u,i}\right)\delta_{u,i,0}\right]+\left(1-y_{u,i}\right)\delta_{u,i,0}.$$

Similarly, the loss of RD-DR, under Assumptions 1, 3 and 4 for the PU learning framework, is defined as

$$\begin{array}{cc} L_{RD-DR}\; & =\max_{W\in W}\frac{1}{\left|D\right|}\sum_{(u,i)\in D}y_{u,i}\left[w_{u,i}\left(\delta_{u,i,1}-\delta_{u,i}^{imp}\right)+\left(1-w_{u,i}\right)\left(\delta_{u,i,0}-\delta_{u,i}^{imp}\right)\right]\\ \; & \;+\left(1-y_{u,i}\right)\left(\delta_{u,i,0}-\delta_{u,i}^{imp}\right)+\delta_{u,i}^{imp} \end{array}$$

Intuitively, the RD-IPS/DR losses are robust to unmeasured confounders by controlling the worst-case scenario induced by unmeasured confounders. Moreover, a similar strategy can be applied to any propensity-based methods, such as DR-JL [37], MRDR [38], DR-MSE [24], etc.

We now analyze the generalization bounds of RD-IPS and RD-DR loss functions. Let H be the finite hypothesis space of the prediction model $\hat{r}$. For clarity, for any prediction model ${\hat{r}}^{h}\in H$, we write $L_{ideal}$ as $L_{ideal}\left({\hat{r}}^{h}\right)$ to highlight its dependence on ${\hat{r}}^{h}$. Similarly, we denote $L_{RD-IPS}=L_{RD-IPS}\left({\hat{r}}^{h}\right)$ and $L_{RD-DR}=L_{RD-DR}\left({\hat{r}}^{h}\right)$.

**Theorem** **3.** *Suppose that ${\tilde{w}}_{u,i}\in \left[{\hat{a}}_{u,i},{\hat{b}}_{u,i}\right]$, $\delta_{u,i}\leq C_{1}$, and ${\tilde{w}}_{u,i}\leq C_{2}$. Then for any prediction model ${\hat{r}}^{h}\in H$ and η>0, we have a probability of at least 1−η,*

$$\begin{array}{cc} L_{ideal}\left({\hat{r}}^{h}\right)\leq & L_{RD-IPS}\left({\hat{r}}^{h}\right)+C_{1}\left(C_{2}+1\right)\sqrt{\frac{2\log(|H|/\eta )}{\left|D\right|}}. \end{array}$$

*In addition, given the imputed error $\delta_{u,i}^{imp}$ and assume that $|\delta_{u,i}-\delta_{u,i}^{imp}|\;\leq C_{3}$, then with a probability of at least 1−η,*

$$\begin{array}{cc} L_{ideal}\left({\hat{r}}^{h}\right)\leq & L_{RD-DR}\left({\hat{r}}^{h}\right)+C_{3}\left(C_{2}+1\right)\sqrt{\frac{2\log(|H|/\eta )}{\left|D\right|}}. \end{array}$$

**Proof.**  For any prediction model ${\hat{r}}^{h}\in H$, since ${\tilde{w}}_{u,i}\in \left[{\hat{a}}_{u,i},{\hat{b}}_{u,i}\right]$, we have

$$\begin{array}{cc} \; & L_{ideal}\left({\hat{r}}^{h}\right)-L_{RD-IPS}\left({\hat{r}}^{h}\right)\\ \leq & L_{ideal}\left({\hat{r}}^{h}\right)-\frac{1}{\left|D\right|}\sum_{(u,i)\in D}y_{u,i}\left[{\tilde{w}}_{u,i}\left(\delta_{u,i,1}-\delta_{u,i}^{imp}\right)+\left(1-{\tilde{w}}_{u,i}\right)\left(\delta_{u,i,0}-\delta_{u,i}^{imp}\right)\right]\\ \; & \;-\left(1-y_{u,i}\right)\left(\delta_{u,i,0}-\delta_{u,i}^{imp}\right)+\delta_{u,i}^{imp}\\ = & \frac{1}{\left|D\right|}\sum_{(u,i)\in D}\left({\hat{r}}_{u,i}^{h}-y_{u,i}{\tilde{w}}_{u,i}\right)\delta_{u,i,1}+\left(y_{u,i}{\tilde{w}}_{u,i}-{\hat{r}}_{u,i}^{h}\right)\delta_{u,i,0}. \end{array}$$

Under Assumption 3, the expectation of $\left(\delta_{u,i,1}-\delta_{u,i,0}\right)\left({\hat{r}}_{u,i}^{h}-y_{u,i}{\tilde{w}}_{u,i}\right)$ is 0. Note that $|\delta_{u,i,y_{u,i}}\left({\hat{r}}_{u,i}^{h}-y_{u,i}{\tilde{w}}_{u,i}\right)|\;\leq C_{1}\left(C_{2}+1\right)$, and $\hat{\delta },r^{h}\in \left\{0,1\right\}$. Applying Hoeffding’s inequality yields the following result,

$$P\left\{\frac{1}{\left|D\right|}\sum_{(u,i)\in D}\left({\hat{r}}_{u,i}^{h}-y_{u,i}{\tilde{w}}_{u,i}\right)\left(\delta_{u,i,1}-\delta_{u,i,0}\right)>\epsilon \right\}\leq \exp\left\{-\frac{\epsilon^{2}\left|D\right|}{2C_{1}^{2}{\left(C_{2}+1\right)}^{2}}\right\}.$$

Then

$$\begin{array}{cc} \; & P\left\{L_{ideal}\left({\hat{r}}^{h}\right)-L_{RD-IPS}\left({\hat{r}}^{h}\right)\leq \epsilon \right\}\\ = & 1-P\left\{L_{ideal}\left({\hat{r}}^{h}\right)-L_{RD-IPS}\left({\hat{r}}^{h}\right)>\epsilon \right\}\\ \geq & 1-P\left\{\underset{{\hat{y}}^{h}\in H}{\sup}\left[L_{ideal}\left({\hat{r}}^{h}\right)-L_{RD-IPS}\left({\hat{r}}^{h}\right)\right]>\epsilon \right\}\\ \geq & 1-\sum_{h=1}^{\left|H\right|}P\left\{L_{ideal}\left({\hat{r}}^{h}\right)-L_{RD-IPS}\left({\hat{r}}^{h}\right)>\epsilon \right\}\\ \geq & 1-\sum_{h=1}^{\left|H\right|}P\left\{\frac{1}{\left|D\right|}\sum_{(u,i)\in D}\left({\hat{r}}_{u,i}^{h}-y_{u,i}{\tilde{w}}_{u,i}\right)\left(\delta_{u,i,1}-\delta_{u,i,0}\right)>\epsilon \right\}\\ \geq & 1-|H|\exp\left\{-\frac{\epsilon^{2}\left|D\right|}{2C_{1}^{2}{\left(C_{2}+1\right)}^{2}}\right\}. \end{array}$$

Letting $|H|\exp\{-\epsilon^{2}|D|/2C_{1}^{2}{\left(C_{2}+1\right)}^{2}\}=\eta$ leads to

$$\epsilon =C_{1}\left(C_{2}+1\right)\sqrt{\frac{2\log(|H|/\eta )}{\left|D\right|}}.$$

Thus, with a probability of at least 1−η,

$$\begin{array}{cc} L_{ideal}\left({\hat{r}}^{h}\right)\leq & L_{RD-IPS}\left({\hat{r}}^{h}\right)+C_{1}\left(C_{2}+1\right)\sqrt{\frac{2\log(|H|/\eta )}{\left|D\right|}}. \end{array}$$

Next, for the generalization error bound of RD-DR. Observe that

$$\begin{array}{cc} \; & L_{ideal}\left({\hat{r}}^{h}\right)-L_{RD-DR}\left({\hat{r}}^{h}\right)\\ \leq & L_{ideal}\left({\hat{r}}^{h}\right)-\sum_{(u,i)\in D}y_{u,i}\left[\frac{1}{{\hat{p}}_{u,i}}\left(\delta_{u,i,1}-\delta_{u,i}^{imp}\right)+\left(1-\frac{1}{{\hat{p}}_{u,i}}\right)\left(\delta_{u,i,0}-\delta_{u,i}^{imp}\right)\right]\\ \; & \;\;\;\;\;\;\;\;+\left(1-y_{u,i}\right)\left(\delta_{u,i,0}-\delta_{u,i}^{imp}\right)+\delta_{u,i}^{imp}\\ = & \frac{1}{\left|D\right|}\sum_{(u,i)\in D}\left({\hat{r}}_{u,i}^{h}-y_{u,i}{\tilde{w}}_{u,i}\right)\left(\delta_{u,i,1}-\delta_{u,i}^{imp}\right)+\left(y_{u,i}{\tilde{w}}_{u,i}-{\hat{r}}_{u,i}^{h}\right)\left(\delta_{u,i,1}-\delta_{u,i}^{imp}\right). \end{array}$$

Given the imputed error $\delta_{u,i}^{imp}$ and assume that $|\delta_{u,i}-\delta_{u,i}^{imp}|\;\leq C_{3}$ (upper bound exists since δ∈[0,1]), the expectation of $\left(\delta_{u,i}-\delta_{u,i}^{imp}\right)\left({\hat{r}}_{u,i}^{h}-y_{u,i}{\tilde{w}}_{u,i}\right)$ is 0 under Assumption 3 and $|\left(\delta_{u,i}-\delta_{u,i}^{imp}\right)\left({\hat{r}}_{u,i}^{h}-y_{u,i}{\tilde{w}}_{u,i}\right)|\;\leq C_{3}\left(C_{2}+1\right)$. Then by exactly the same arguments as the proof of IPS, we obtain that with probability at least 1−η,

$$\begin{array}{cc} L_{ideal}\left({\hat{r}}^{h}\right)\leq & L_{RD-DR}\left({\hat{r}}^{h}\right)+C_{3}\left(C_{2}+1\right)\sqrt{\frac{2\log(|H|/\eta )}{\left|D\right|}}. \end{array}$$

□

Theorem 3 holds for any ${\hat{r}}^{h}$, including ${\hat{r}}^{h*}\left(1\right)$ and ${\hat{r}}^{h†}\left(1\right)$ defined by $r^{h*}\left(1\right)=\arg min_{{\hat{r}}^{h}}L_{RD-IPS}\left({\hat{r}}^{h}\right)$ and ${\hat{r}}^{h†}\left(1\right)=\arg min_{{\hat{r}}^{h}}L_{RD-DR}\left({\hat{r}}^{h}\right)$. It shows that the generalization bound of the prediction model trained by the RD-IPS or RD-DR loss is bounded by its corresponding loss plus a negligible term. The negligible term vanishes at a rate of $O(1/\sqrt{\left|D\right|})$ as |D| goes to infinity, which suggests that the loss of RD-IPS or RD-DR itself can well control the generalization bounds, even in the presence of unobserved confounders. This theoretically demonstrates the effectiveness of the proposed method.

Overall, the structural design of the proposed two-phase framework clarifies how the estimator operates under the presence of missing exposures and unmeasured confounding. The treatment-imputation module reconstructs latent exposure patterns by identifying user–item pairs that align geometrically with the observed logging mechanism by SVDD one-class classification, thereby producing an expanded training sample that reflects the selection dynamics of the underlying system. Although such reconstruction may introduce moderate discrepancies relative to the true exposure distribution, the subsequent debiasing phase explicitly regulates the influence of unmeasured confounding through bounded inverse-propensity adjustments. These bounds determine how uncertainty in the exposure mechanism propagates into the weighted optimization objective and compensates for potential inaccuracies created during imputation. Conversely, the debiasing phase benefits from the imputation phase because effective correction requires initial propensity values that retain meaningful structure from the logged data rather than being dominated by unobserved variation. The two phases thus form a complementary procedure in which the imputation step provides informative starting values and the debiasing step refines them to achieve a controlled adjustment of both exposure noise and hidden confounding. Since both phases operate at the level of exposure modeling and weighting rather than depending on specific prediction architectures, the framework integrates seamlessly with those propensity-based estimators originally designed for partially observed explicit feedback, yielding a coherent and transparent estimation pipeline.

## 5. Experiments

In this section, several experiments are conducted on real datasets to verify the efficiency of the proposed two-phase implicit feedback debiasing framework, which combines SVDD for treatment imputation and adversarial learning for debiasing. We aim to answer the following research questions (RQs):

**RQ1.**  Does the proposed method outperform the existing debiasing methods on real-world datasets?
**RQ2.**  How does each of the stages affect the debiasing performance of the proposed method?
**RQ3.**  Does our method stably outperform for different SVDD sampling ratios on positive treatment imputations?
**RQ4.**  How does the adversarial strength affect the debiasing performance?
**RQ5.**  Given the model complexity, is there a potential overfitting issue?

### 5.1. Experimental Setup

**Datasets.** Measuring the debiasing performance requires that the dataset contains both missing-not-at-random (MNAR) ratings and missing-at-random (MAR) ratings. There are three public datasets that meet the requirements. The first is **Coat Shopping** (https://www.cs.cornell.edu/~schnabts/mnar/, accessed on 3 November 2025), which contains 6960 MNAR ratings and 4640 MAR ratings in total. Both MNAR ratings and MAR ratings are generated by 290 users to 300 items. Each user rates their favorite 24 products to generate the former, while each user randomly rates 16 products to make up of the latter. The second is **Yahoo! R3** (https://www.kaggle.com/datasets/limitiao/yahoor3, accessed on 3 November 2025), which contains ratings from 15,400 users to 1000 items. Each user rates several items to generate the 311,704 MNAR ratings, while the first 5400 users are asked to randomly rate 10 items, which make up the 54,000 MAR ratings. The third is KuaiRec (https://kuairec.com/, accessed on 3 November 2025), a public large-scale dataset, collected via a video-sharing platform, which consists of 4,676,570 video watching ratio records from 1411 users and 3327 videos. To further demonstrate the generalizability of our method performance, we utilize its unbiased data, where a subset of users is asked to rate sampled items uniformly at random, providing 13,454 MAR ratings, leaving 201,171 interactions to serve as MNAR data.
**Pre-processing.** The difference between the rating prediction task in implicit and explicit feedback settings is that the implicit feedback cannot see the rating if the user does not like the item. Following previous studies [37,38,59], we binarize the ratings as negative if the rating is less than 3, otherwise as positive. Therefore, we remove the samples with ratings less than 3 from three datasets, leaving 3622 positive samples for the Coat dataset, 174,208 positive samples for the Yahoo! R3 dataset, and 105,186 positive samples for the KuaiRec dataset.
**Baselines.** To validate the efficiency of the proposed debiasing framework, we compare our methods with the following baselines:

- **Base model**: **Matrix Factorization (MF)** [77] is used as the base model frequently in debiasing recommendations.
- **WMF**: WMF [15] is a classic method for implicit feedback. It up-weights the loss of all positive feedback to reflect greater confidence in the positive feedback than the negative feedback.
- **BPR**: BPR [42] is another classic method for implicit feedback. It assumes that unclicked items are less preferred than clicked items and, therefore, maximizes the posterior probability under this assumption.
- **Rel-MF**: Rel-MF [14] is a debiasing method for implicit feedback, which is an extension of the classic IPS methods [22] for implicit feedback.
- **IPS** [22]: IPS is a classic debiasing method that weights the loss function by the corresponding inverse propensity scores to reduce the bias.
- **DR** [59]: DR is another classic debiasing method that includes both the propensity and the imputation model and aims to minimize the DR loss to reduce bias.
- **DR-JL** [37]: DR-JL is based on the DR method. It applies a joint learning algorithm between the prediction and the imputation model.
- **MRDR-JL** [38]: MRDR-JL is a variation of DR-JL, which changes the original DR imputation loss to MRDR imputation loss.

**Negative Sampling for Baselines.** Note that the existing methods require training with both positive samples and negative samples. However, we can only observe positive samples in the implicit feedback setting. Thus, we use the negative sampling method with the baseline methods. Specifically, we randomly select user–item pairs in the missing data and mark them as negative samples, and the estimated propensities are obtained by logistic regression.
**Experimental protocols and details.** Following the previous studies [22,60], MSE, AUC, NDCG@5 and NDCG@10 are used as our evaluation metrics. All experiments are implemented on PyTorch v2.6.0 with Adam as the optimizer (for all experiments, we use GeForce RTX 2060 as the computing resource). To mitigate overfitting, we employ L2 regularization via weight decay and early stopping during training, which is terminated when the relative loss decrease falls below a $1\times 10^{-4}$ tolerance threshold for 5 consecutive epochs. We tune the weight decay in $\left\{1\times 10^{-5},2.5\times 1\times 10^{-5},5\times 1\times 10^{-5},\ldots ,7.5\times 1\times 10^{-3},1\times 10^{-2}\right\}$ and the learning rate in {0.005,0.01,0.05,0.1}. In addition, we tune the sample ratio between the imputed negative and positive samples in {0.6,0.8,1.0,1.2,1.4} in RQ3 and set it to 1.0 for other RQs. For the RD-based methods, we tune the adversarial strength gamma in {1.0,1.05,1.1,1.15,1.2} in RQ4 and set it to 1.1 for other RQs. We set the batch size to 128 for **Coat**, 2048 for **Yahoo! R3 and KuaiRec**, and use the default parameter values in the SVDD function in scikit-learn v1.3.0 package for the SVDD-based methods on both datasets.

### 5.2. Real-World Performance (RQ1)

We conducted experiments on three real datasets, **Coat**, **Yahoo! R3** and **KuaiRec**, to verify the effectiveness of the proposed two-phase debiasing framework for implicit feedback. Table 1 compares the previous debiasing methods and their prediction performance when combined with the two-phase debiasing framework. Since the Naive method simply uses observed samples without estimating propensities, it cannot be combined with RD for unmeasured confounders. On the one hand, the models combined with the debiasing method all significantly outperform the Naive method, which illustrates the necessity of introducing the debiasing method, as well as the effectiveness and flexibility of the proposed debiasing framework for combining with various debiasing methods in explicit feedback. On the other hand, when dealing with the implicit feedback problem, we found that the debiasing method combining both phases can significantly improve the prediction performance in all metrics compared to the original debiasing methods used in explicit feedback, which is explained by the fact that the treatment imputation in the first stage can help the estimation of propensities and the accurate imputation of negative samples, while the second stage can further adjust for the unmeasured confounders using an RD technique based on the information provided in the first stage.

To ensure a direct and fair evaluation focused on debiasing performance, we follow the established practice in the debiasing literature [14] by employing MF as the predictive model backbone for all primary comparisons. This provides a consistent benchmark to isolate and evaluate the efficacy of the debiasing components. To further verify the robustness of our framework across different representation learners, we conducted an exploratory analysis using the VAE-CF method (Variational Autoencoder for Collaborative Filtering [78]) as an alternative on the Coat dataset. The MRDR-JL-based results are presented in Appendix B, Table A1. They indicate that the relative performance improvements achieved by our two-stage debiasing framework are maintained when using such advanced architecture, demonstrating its general applicability.

### 5.3. Ablation Study (RQ2)

We further perform an ablation study to investigate the effect of each phase on debiasing performance in implicit feedback. As shown in Figure 1, across all baseline methods, adding either of the two phases can significantly improve performance on MSE, AUC, and NDCG@K. For phase 1, it should be noted that the proposed SVDD uses positive sampling for treatment, rather than direct negative sampling of ratings as suggested by previous studies. This has the advantage that treatment imputation not only provides a more accurate estimate of propensities but also provides accurate inferences for the relevance of interest, i.e., for negative samples $y_{u,i}=0$, the additional information of $o_{u,i}=1$ obtained by phase 1 can lead to the conclusion of $r_{u,i}=0$. In addition, the SVDD algorithm can yield more convincing positive treatment samples than the previous random negative sampling method for the labeling of $r_{u,i}=0$. For phase 2, by incorporating RD, not only can the direct effect of unmeasured confounders on propensity estimation be adjusted using adversarial techniques but also the reduction in debiasing performance due to inaccurate treatment imputation in phase 1 can be mitigated. Optimal prediction performance is achieved when both debiasing phases are combined, due to the simultaneous use of treatment imputation for propensity estimation and relevance prediction, as well as the adjustment of unmeasured confounders using RD.

### 5.4. In-Depth Analysis (RQ3 and RQ4)

**Figure 3:**
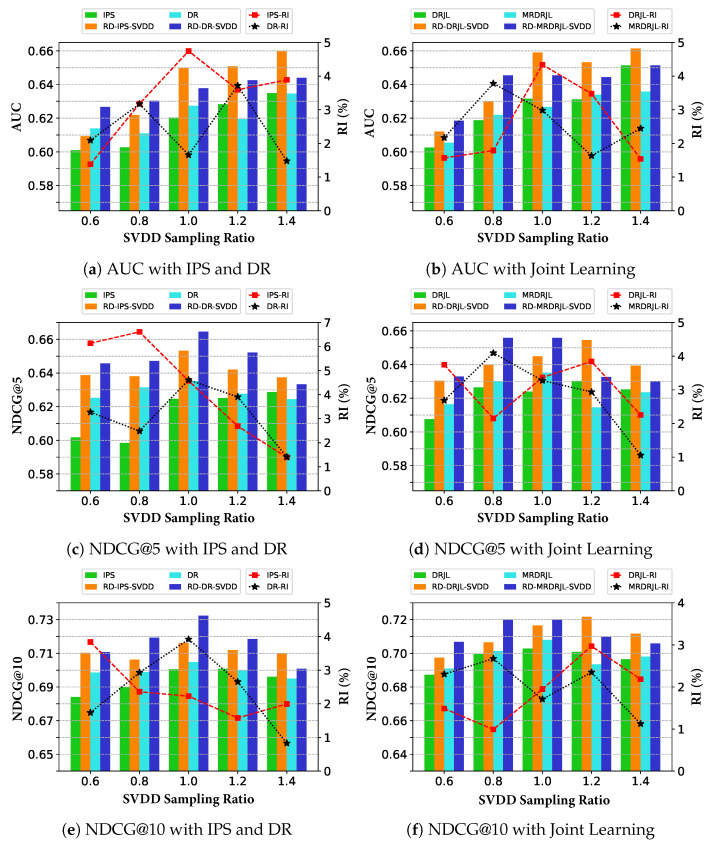
The effect of an SVDD treatment imputation positive sampling ratio on AUC, NDCG@5, and NDCG@10 for the proposed two-phase debiasing framework.

**Figure 4:**
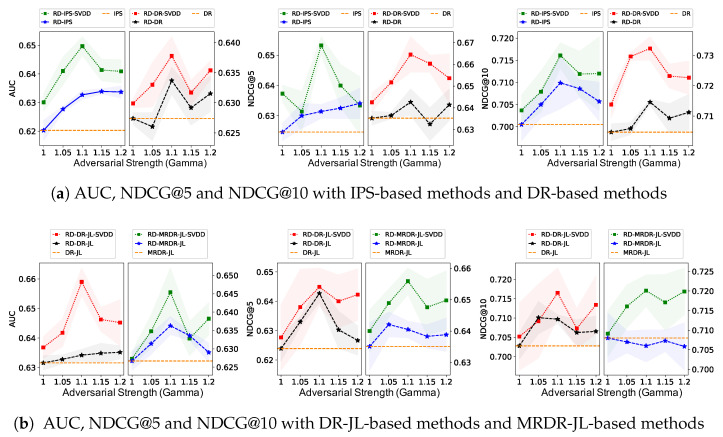
AUC, NDCG@5 and NDCG@10 on MAR data with the different adversarial strength. The above are the IPS and DR methods and the bottom are the DR-based methods with the joint learning algorithm.

**Effect of SVDD Sampling Ratio in the First Stage.** In the first stage, SVDD can provide more reliable imputations for positive treatment, leading to more accurate estimation of propensities and relevance predictions. Figure 3 demonstrates the effect of the various debiasing methods on AUC, NDCG@5, and NDCG@10 at different sample ratios of positive treatment imputations. Remarkably, the proposed method outperforms significantly over the baseline with all SVDD sampling ratios. In addition, predictive performance reaches an optimal for proper positive sampling ratios between 0.8 and 1.2. This is explained by the fact that too many or too few imputations of positive treatment can cause data imbalance, and the positive imputations by the SVDD method result in lower confidence as the number of imputations increases.
**Sensitivity of Adversarial Strength in the Second Stage.** For the second stage, we conducted repeated experiments to investigate the effect of different adversarial strengths of RD on the AUC, NDCG@5, and NDCG@10 of the various debiasing methods, and the results are shown in Figure 4, where the yellow dashed line shows the performance of the baseline method without RD. The proposed method stably outperforms the baselines for almost all adversarial strengths, validating the effectiveness of RD’s tuning for unmeasured confounders. In addition, a higher adversarial strength increases the variance, and a suitable adversarial strength between 1.05 and 1.15 leads to optimal performance. Such empirical results are compatible with the theoretical guarantee, where a larger adversarial strength not only has a stronger effect on the adjustment of unmeasured confounders but also increases the hypothesis space of the prediction model, leading to a higher variance as well as worsening the performance of the model given by the minimax method. When the adversarial strength is set to 1, it is equivalent to not adjusting for unmeasured confounders, which degenerates to debiasing methods without using RD techniques.

### 5.5. Overfitting Analysis (RQ5)

**Figure 5:**
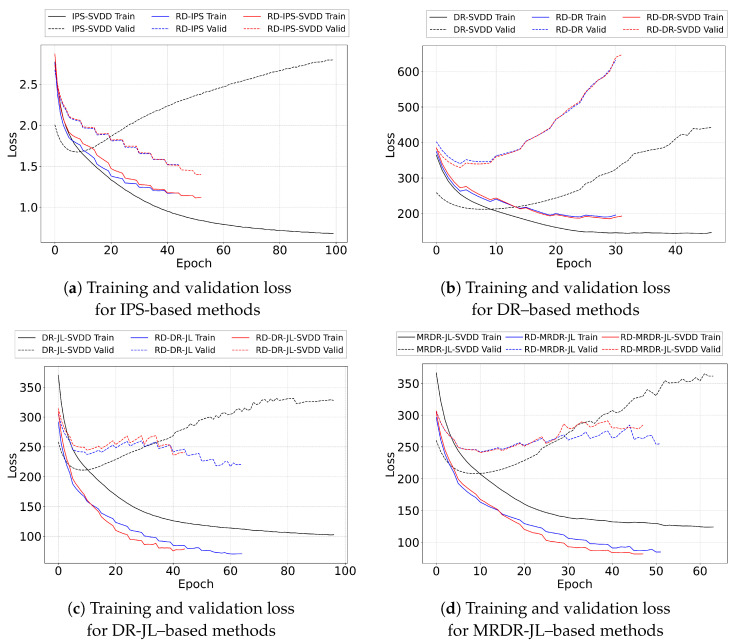
Training and validation loss curves for SVDD-only, RD-only, and RD-SVDD variants of the IPS, DR, DR-JL, and MRDR-JL estimators. The RD-SVDD configuration provides the most stable convergence across families. In the DR family, intrinsic variance of the DR objective produces more pronounced overfitting, though RD-SVDD remains more stable than RD-only.

**Overfitting Analysis.** We evaluate whether the proposed two-phase debiasing framework leads to overfitting by tracking the IPS-weighted training and validation losses across epochs. The trajectories in Figure 5 show that SVDD-only variants within the IPS, DR-JL, and MRDR-JL families converge slowly and display clear upward trends in validation loss, reflecting that treatment imputation alone increases data coverage but does not correct the confounding structure of the logged data, making the models sensitive to pseudo-negative noise. Adding the RD phase mitigates this issue: RD-only variants converge more quickly and exhibit noticeably reduced overfitting, which is consistent with RD’s ability to regulate propensity adjustments and limit the influence of unmeasured confounders. The full two-phase method combining SVDD and RD further stabilizes the trajectories, reaching convergence more rapidly and maintaining lower validation losses near convergence.

The DR family shows a different pattern. Although RD-only and RD-SVDD variants converge faster than their SVDD-only counterparts, both exhibit larger increases in validation loss in later epochs. This stems from the DR objective’s separate estimation of propensity weighting and imputation components, where small mismatches between the two accumulate and amplify variance, making DR more sensitive to noisy pseudo-negatives than DR-JL or MRDR-JL. Nevertheless, RD-SVDD remains more stable than RD-only, indicating that incorporating treatment imputation continues to provide benefit within the DR family.

Overall, the loss trajectories demonstrate that the SVDD-RD framework and its DR-JL and MRDR extensions do not induce harmful overfitting. Since this loss corresponds directly to the optimization objective, the close agreement between training and validation losses shows that the proposed methods generalize reliably despite their increased structural complexity.

**Runtime Analysis.** In addition, we investigate the potential computational overhead issue and conduct a comprehensive runtime analysis across all three datasets. The total training time for each debiasing method is summarized in Table 2. A key observation is that our complete two-phase methods, such as RD-IPS-SVDD, RD-DR-SVDD, exhibit training times comparable to their base single-phase or non-robust counterparts, attributed to the stabilized training dynamics and early stopping. The results demonstrate that the superior prediction performance reported in Table 1 is achieved with a measurable yet manageable computational overhead.

## 6. Conclusions

The positive-unlabeled (PU) learning problem appears widely in modern machine learning tasks, including computer vision, natural language processing, and recommender systems, where the lack of explicit negative labels and the missing-not-at-random (MNAR) nature of feedback make unbiased estimation difficult. This work proposes a two-phase debiasing framework that addresses both missing exposure and unmeasured confounding and extends classical propensity-based estimators to PU settings. In the first phase, exposure imputation is formulated as a one-class classification problem, where SVDD provides quantitative signals describing how unlabeled entries relate to the observed logging mechanism. In the second phase, a robust deconfounding component introduces bounded propensity adjustments to correct for unmeasured confounders and to adjust for errors propagated from the imputation step. The resulting RD-version loss estimators restore unbiasedness under weakened assumptions and are supported by generalization guarantees that reflect the controlled impact of hidden confounding. Together, the two phases form a coherent procedure in which surrogate exposure reconstruction and confounding adjustment act through explicit and structurally defined components, well aligned with the PU learning problem.

In practical deployment, this framework is most effective where the observed labels reflect the true preference. The foundational y=o·r assumption can be violated by label noise, but we could still presume it holds for the true labels, which may not be able to be observed in this scenario. Extending the framework to jointly achieve debiasing and denoising, such as the approach of identifying the noise label transition matrix, is a valuable direction for future research. Learning the robustness parameter Γ for unmeasured confounding remains another practical question. One promising direction is by fusion with a small unbiased dataset, which is available in three real-world datasets. Moreover, the performance of our method relies on having sufficient data for reliable exposure imputation. Even though the DR estimator that our method is based on provides theoretical guarantees of double robustness, i.e., unbiased estimation if either imputation error or outcome is well predicted, the accuracy of the exposure prediction still influences the estimation variance.

Experiments on three real-world datasets verify the effectiveness of this design. Across classic propensity-based methods, adding either SVDD-based exposure reconstruction or RD-based adjustment improves performance, and combining both consistently yields the strongest results. The ablation analyses show that SVDD offers more informative exposure imputations than random negative sampling, while RD stabilizes weighting and reduces noise introduced during imputation. Sensitivity studies demonstrate that moderate SVDD sampling ratios and moderate RD adversarial strengths provide the best balance between reconstruction accuracy and variance control. Training–validation loss trajectories further confirm that the full two-phase variants converge efficiently and resist overfitting.

Taken together, this work contributes a general, theoretically grounded, and practically effective framework for PU learning that integrates exposure modeling and confounding adjustment into a unified estimation pipeline. Beyond implicit-feedback recommendation, the modular structure of the two phases allows the framework to be combined with a broad class of debiasing objectives and prediction models in PU settings where exposure uncertainty and unmeasured confounding coexist. The results highlight the importance of combining the treatment reconstruction with the debiasing step and demonstrate how this joint consideration yields predictable behavior, improved robustness, and consistent gains across metrics and datasets.

## Figures and Tables

**Table 1:** MSE, AUC, NDCG@5 (N@5), and NDCG@10 (N@10) on the MAR data of Coat, Yahoo! R3, and KuaiRec. We bold the outperforming IPS-based, DR-based, and MRDR-based models.

Method	Coat				Yahoo!R3				KuaiRec			
**Metrics**	**MSE**	**AUC**	**N@5**	**N@10**	**MSE**	**AUC**	**N@5**	**N@10**	**MSE**	**AUC**	**N@5**	**N@10**
Base Model (MF)	0.2414	0.6038	0.6294	0.6998	0.2089	0.6329	0.7104	0.8062	0.1837	0.8095	0.5315	0.5498
+ WMF	0.2447	0.6289	0.6356	0.7060	0.2105	0.6487	0.7279	0.8174	0.1818	0.8180	0.5348	0.5544
+ BPR-MF	0.2492	0.6136	0.6378	0.7061	0.2057	0.6433	0.7102	0.8074	0.1848	0.8178	0.5342	0.5447
+ Rel-MF	0.2404	0.6312	0.6368	0.7025	0.2128	0.6655	0.7138	0.8069	0.1786	0.8204	0.5357	0.5486
+ IPS	0.2350	0.6203	0.6246	0.7005	0.2034	0.6328	0.7053	0.8050	0.1752	0.8133	0.5130	0.5374
+ IPS-SVDD	0.2339	0.6301	0.6373	0.7037	0.2116	0.6641	0.7267	0.8152	0.1733	0.8205	0.5269	0.5472
+ RD-IPS	0.2328	0.6327	0.6314	0.7099	0.2097	0.6658	0.7215	0.8125	0.1728	0.8193	0.5295	0.5521
+ RD-IPS-SVDD	**0.2280**	**0.6497**	**0.6532**	**0.7161**	**0.1934**	**0.6663**	**0.7297**	**0.8184**	**0.1691**	**0.8265**	**0.5401**	**0.5603**
+ DR	0.2473	0.6274	0.6352	0.7047	0.2049	0.6379	0.7161	0.8110	0.1775	0.8305	0.5337	0.5541
+ DR-SVDD	**0.2405**	0.6299	0.6425	0.7138	0.2054	0.6565	0.7252	0.8166	**0.1757**	0.8287	0.5357	0.5533
+ RD-DR	0.2438	0.6337	0.6426	0.7146	0.2057	0.6675	0.7227	0.8144	0.1787	0.8315	0.5371	0.5530
+ RD-DR-SVDD	0.2410	**0.6378**	**0.6645**	**0.7323**	**0.1998**	**0.6706**	**0.7358**	**0.8224**	0.1777	**0.8324**	0.5458	**0.5544**
+ DR-JL	0.2339	0.6316	0.6239	0.7028	0.2024	0.6329	0.7066	0.8050	0.1761	0.8182	0.5206	0.5369
+ DR-JL-SVDD	0.2326	0.6368	0.6277	0.7052	0.2036	0.6346	**0.7166**	0.8105	0.1736	0.8160	**0.5232**	0.5444
+ RD-DR-JL	0.2324	0.6342	0.6427	0.7097	0.1976	0.6419	0.7098	0.8064	0.1761	0.8182	0.5223	0.5463
+ RD-DR-JL-SVDD	**0.2288**	**0.6590**	**0.6449**	**0.7165**	**0.1958**	**0.6477**	**0.7166**	**0.8122**	**0.1733**	**0.8197**	**0.5232**	**0.5464**
+ MRDR-JL	0.2367	0.6267	0.6351	0.7080	0.2037	0.6313	0.7077	0.8062	0.1770	0.8142	0.5238	0.5394
+ MRDR-JL-SVDD	0.2350	0.6273	0.6400	0.7091	0.1992	0.6377	0.7165	0.8116	0.1759	0.8166	0.5302	0.5489
+ RD-MRDR-JL	0.2333	0.6363	0.6405	0.7060	0.2013	0.6369	0.7197	0.8127	0.1757	0.8163	0.5257	0.5387
+ RD-MRDR-JL-SVDD	**0.2288**	**0.6454**	**0.6559**	**0.7201**	**0.1990**	**0.6466**	**0.7217**	**0.8144**	**0.1689**	**0.8190**	**0.5331**	**0.5557**

**Table 2:** Training duration (seconds) comparison on Coat, Yahoo!R3 and KuaiRec datasets.

Method	Coat	Yahoo!R3	KuaiRec
MF	7.61	15.31	8.62
MF-SVDD	4.37	9.59	4.71
IPS	7.85	8.23	13.35
IPS-SVDD	6.28	11.44	11.42
RD-IPS	3.54	59.66	51.84
RD-IPS-SVDD	4.48	58.09	54.31
DR	4.28	50.89	29.26
DR-SVDD	4.81	57.08	26.30
RD-DR	4.32	104.22	29.24
RD-DR-SVDD	4.37	99.02	29.88
DR-JL	10.99	74.61	88.13
DR-JL-SVDD	20.58	68.04	69.75
RD-DR-JL	16.90	125.52	125.16
RD-DR-JL-SVDD	12.14	132.56	114.56
MRDR-JL	10.63	68.97	47.56
MRDR-JL-SVDD	13.89	79.24	78.00
RD-MRDR-JL	17.20	185.23	165.26
RD-MRDR-JL-SVDD	12.93	189.68	157.06

## Data Availability

The data that support the findings of this study are publicly available from open-access sources, including the Coat Shopping dataset accessed via https://www.cs.cornell.edu/~schnabts/mnar/ (accessed on 3 November 2025), the Yahoo! R3 dataset available at https://www.kaggle.com/datasets/limitiao/yahoor3 (accessed on 3 November 2025), and the KuaiRec dataset openly accessible through https://kuairec.com/.

## Appendix A. Proofs

**Proof of Theorem** **1.** It suffices to show that Equation (9) does not hold under Assumption 3. This follows immediately from the truth that $r_{u,i}$ is associated with $o_{u,i}$ given $x_{u,i}$ due to the existence of unmeasured confounders $h_{u,i}$ (See Figure 2). □

**Proof of Theorem** **2.** The conclusion of Theorem 2(a) can be found in Theorem 3.1 of [57]. The result of Theorem 2(b) holds since Equation (16) is implied by Assumptions 1 and 3 directly, and Propositions 1 and 2. □

**Proof of Lemma** **1.** By definition, it is easy to see that

(A1) $$m\left(x_{u,i}\right)=\log\frac{p_{u,i}}{1-p_{u,i}},\;\tilde{m}\left(x_{u,i},h_{u,i}\right)=\log\frac{{\tilde{p}}_{u,i}}{1-{\tilde{p}}_{u,i}}.$$

Following the sensitivity analysis logic in [57] and the principle mentioned in [76], we have that $|\tilde{m}\left(x_{u,i},h_{u,i}\right)-m\left(x_{u,i}\right)|\;\leq \log\Gamma$, and

(A2) $$\frac{1}{\Gamma }\leq e^{\tilde{m}\left(x_{u,i},h_{u,i}\right)-m\left(x_{u,i}\right)}\leq \Gamma .$$

Plugging Equation (A1) into (A2) yields that

$$\frac{1}{\Gamma }\leq \frac{p_{u,i}}{1-p_{u,i}}/\frac{{\tilde{p}}_{u,i}}{1-{\tilde{p}}_{u,i}}\leq \Gamma .$$

By setting ${\tilde{w}}_{u,i}=1/{\tilde{p}}_{u,i}$ and after some simple algebra, we get that

$$1+\left(1/p_{u,i}-1\right)/\Gamma \leq {\tilde{w}}_{u,i}\leq 1+\left(1/p_{u,i}-1\right)\Gamma .$$

□

## Appendix B. Exploratory Experiment

**Table A1:** Exploratory results of MRDR-JL-based methods with VAE-CF backbone on the coat dataset. Best performance in bold.

Method	MSE	AUC	NDCG@5	NDCG@10
+ MRDR-JL	0.2312	0.6324	0.6397	0.7078
+ MRDR-JL-SVDD	0.2309	0.6403	0.6432	0.7117
+ RD_MRDR-JL	0.2313	0.6434	0.6577	0.7139
+ RD_MRDR-JL-SVDD	**0.2215**	**0.6521**	**0.6626**	**0.7252**
